# Melanoma and Colorectal Cancer as Second Primary Cancers: A Scoping Review of Their Association and the Underlying Biological, Lifestyle, and Genetic Factors

**DOI:** 10.1007/s12029-024-01114-7

**Published:** 2025-05-31

**Authors:** Sasha Patil, Arunan Jeyakumar, Vinod Gopalan

**Affiliations:** Medical Education & Pathology, School of Medicine & Dentistry, Griffith University, Gold Coast Campus, Gold Coast, QLD 4222 Australia

**Keywords:** Second primary cancers, Cutaneous melanoma, Colorectal cancer, Genetics, Epidemiology

## Abstract

**Purpose:**

Second primary cancers (SPCs) are independent primary cancers that develop separately from pre-existing malignancies, distinct from metastasis or recurrence. This study aims to review the current literature and analyse the association between melanoma and colorectal cancer (CRC), as well as the factors contributing to the development of these SPCs.

**Methods:**

A scoping review was conducted, including 21 independent studies. Patient data from these studies were analysed and reviewed alongside relevant biological and lifestyle factors.

**Results:**

The studies reported standardised incidence ratios (SIRs) for a second primary colorectal cancer (CRC) following a melanoma diagnosis ranging from 0.62 to 1.55, while SIRs for a second primary melanoma following a CRC diagnosis ranged from 0.89 to 1.55. Males exhibited a higher risk of developing either CRC or melanoma as a second primary cancer (SPC). An inverse relationship between age and the development of CRC was observed, with younger individuals having a higher risk. African-American populations demonstrated a higher prevalence of melanoma and CRC as SPCs compared to Caucasian and other racial groups. Lifestyle factors such as alcohol consumption, sun exposure, and the intake of red and processed meats were associated with an increased risk of developing melanoma or CRC. Genetic mutations in KRAS, NRAS, and BRAF were commonly implicated in the development of both melanoma and CRC, while mutations in CDKN2A and BRCA2 were specifically significant in melanoma.

**Conclusion:**

The association between melanoma and CRC incidence was confirmed through analysis of current literature and is influenced by various biological, lifestyle, and genetic factors. Understanding these correlations is crucial for predicting the risk of SPCs and enhancing the follow-up care of melanoma and CRC survivors.

## Introduction

Second primary cancers (SPCs) are defined as new cancers that arise separate from previous or pre-existing cancers, not due to metastasis or recurrence [1]. Previous epidemiological and clinical studies have found these SPCs are multifactorial, with various environmental, lifestyle, and genetic influences [2]. Both cutaneous melanoma and colorectal cancers (CRC) are known to be associated with SPCs [3].

Cutaneous melanoma is a malignant form of skin cancer that develops from uncontrollable replication of melanocytes—the pigment-forming cells in the epidermis. Fair-skinned populations, such as those found in Europe, North America, and Australia/New Zealand, are most at risk of developing melanoma related to increased sun exposure and UV radiation. Incidence in these populations is seen to increase as significantly as 4–6% each year [4]. However, high-risk countries such as Australia have given great importance to skin cancer awareness campaigns. Increased preventative measures and routine skin checks have led to earlier detection and treatment of lesions, decreasing morbidity rates, and overall improved patient survival [5].

CRC is the third most common cancer in the world, with an estimated 1.9 million new cases in 2020 [6]. While genetics may play a significant role, CRC can develop sporadically, with the incidence being higher in those above 50 years of age. The highest incidences of CRC are seen in countries across Europe, North America, and Australia/New Zealand, with rates of 40.6 per 100,000 person-years seen in 2020 [7]. This may be due to modifiable risk factors associated with a “Western lifestyle”, such as alcohol intake and consumption of red and processed meat [8]. However, established national screening programs are now leading to earlier detection of lesions, leading to more favourable outcomes such as increased patient survival [9].

For primary melanomas and CRC detected at earlier stages, the 5-year survival rates are now 98% and 90%, respectively [10]. Due to this early diagnosis and improved survival, SPCs are becoming more common morbidities in these cancer patients [11, 12]. As these SPCs are seen to critically shorten overall survival, preventative and follow-up measures may need to be improved [13]. Both melanoma and CRC are reported to be potential predictors of one another as SPCs [14–17]. However, to the best of our knowledge, no studies currently provide a detailed review of the association between CRC and melanoma, along with relevant clinicopathological parameters and lifestyle factors. This scoping review aims to evaluate the current literature and analyse the association between melanoma and CRC, exploring factors that may contribute to this relationship. Understanding these associated factors could enhance our understanding of the commonalities between melanoma and CRC pathophysiologies, enabling the development of more targeted follow-up guidelines for cancer survivors to reduce the risk of SPC.

## Methods

### Search Strategy

Using PubMed, Embase, and Web of Science databases, relevant research regarding second primary CRC after melanoma or second primary melanoma after CRC were identified. To ensure all relevant articles were identified, broad search terms such as “colorectal cancer”, “melanoma”, and “second or secondary” were used. The reference lists of these studies were also used as additional sources of eligible papers.

### Inclusion Criteria

Articles were eligible if they were written in English, full text, and written from 1990 to 2023 (when the first possible link between CRC and melanoma was suggested). Studies excluded were those not in English, not in full text, or were written before 1990. Only melanoma of the skin was included.

### Data Extraction

**Fig. 1:**
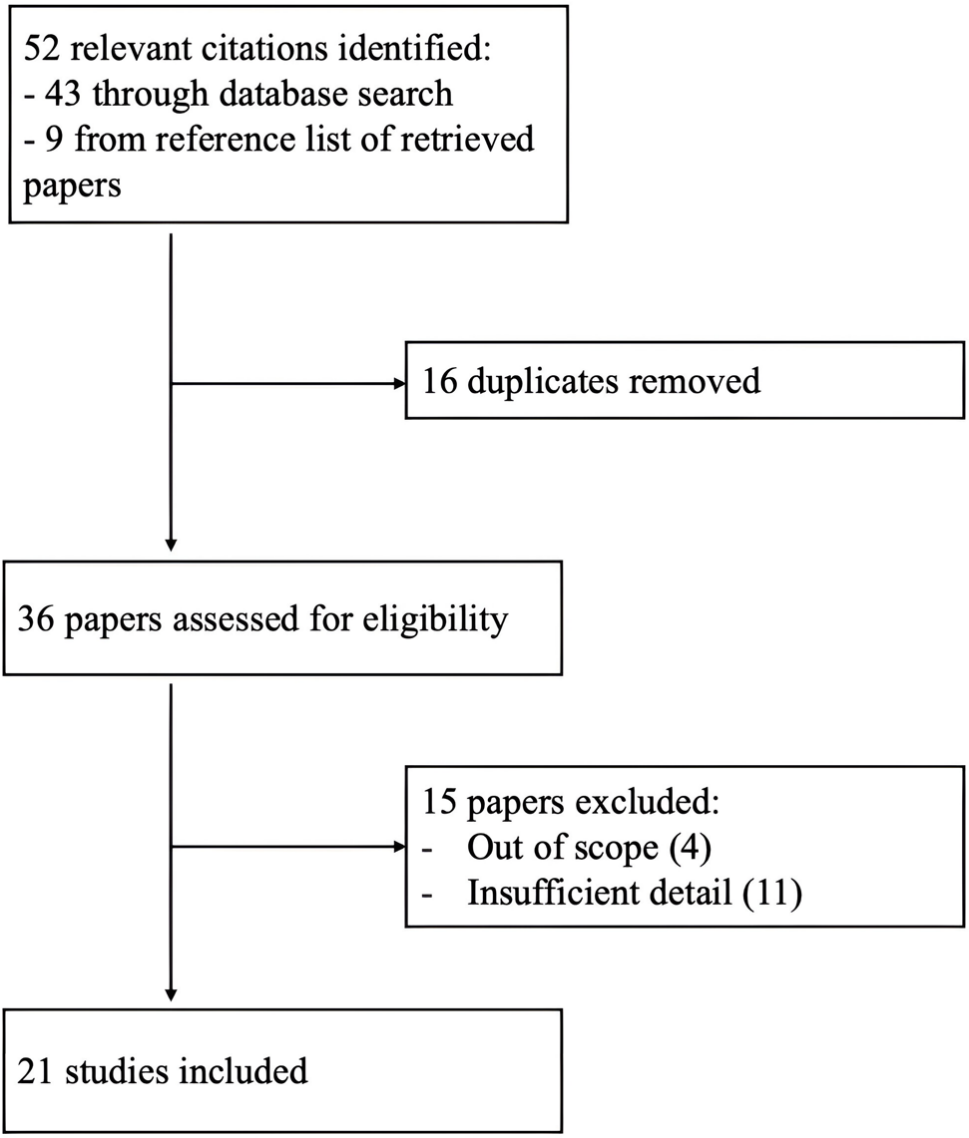
Prisma diagram depicting the selection of included studies

Data was extracted from 21 papers that reported a SIR and summarised into tables. Microsoft Excel was utilised to construct the box and whisker graphs, where the boxes represented the SIR, and the whiskers outlined the confidence interval (CI) Fig. 1.

## Results

### Colorectal Cancer and Its Second Primary Cancers

There have been several studies denoting an association between CRC and the subsequent development of SPCs [16, 18–20]. A meta-analysis study by Robertson et al. included 13 retrospective cohort studies across Europe, Asia, Australia, and North America. With over 7,700,000 CRC patients included, this study noted an overall increased risk of certain extracolonic SPCs compared to the general population’s respective risk [21]. The common SPCs reported were bladder cancers (SIR 1.19, 95% CI 1.06–1.33), female genital tract cancers (1.88, 95% CI 1.07–3.31), renal cancers (1.50, 95% CI 1.19–1.89), thorax (lung, bronchus and mediastinum) cancers (1.16, 95% CI 1.01–1.32), small intestine cancers (4.26, 95% CI 2.58–7.01), stomach cancers (1.22, 95% CI 1.07–1.39), and thyroid cancers (1.40, 95% CI 1.28–1.53). The CRC survivors were also found to have a significantly increased risk of second primary melanoma (1.28, 95% CI 1.01–1.62) [21]. Common genetic/epigenetic changes and environmental exposures were hypothesised as mechanisms of tumorigenesis in these SPCs [21].

#### Melanoma Following Colorectal Cancer

**Fig. 2:**
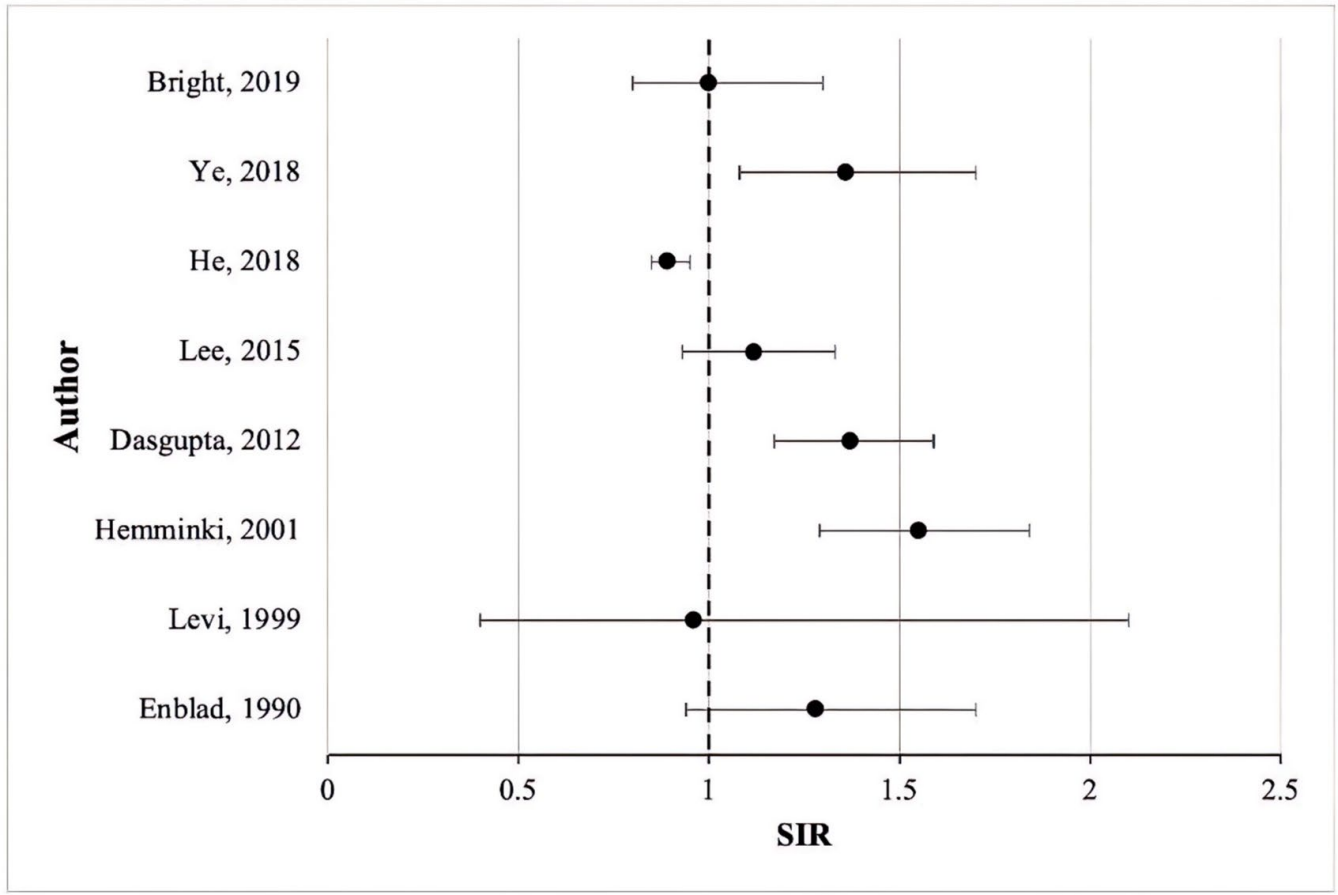
Standardised incidence ratios (SIR) of second primary melanoma following colorectal cancer diagnosis, SIR ranged from 0.89 to 1.55 for primary melanoma after CRC

The occurrence of second primary melanoma in those with a prior diagnosis of CRC is becoming apparent in current literature, with a range of SIR reported (0.89–1.55) (Table 1, Fig. 2). While some studies have reported a decreased risk of melanoma development following CRC (SIR range 0.89–0.96) [19, 22], the majority reported SIRs above one. Although not all these values were statistically significant, the trend suggests an increased risk of developing a second primary melanoma after CRC. However, further research is needed to draw more definitive conclusions regarding this association.

**Table 1:** Studies investigating the risk of second primary melanoma in patients with a previous colorectal cancer diagnosis

Author, year	Data source	No. of CRC patients	Span of study	Melanoma SIR (95% CI)	Key findings
Enblad, 1990 [23]	Swedish Cancer Registry	61,769	1960–1981	1.28 (0.94–1.70)	Melanoma SPC more prevalent in rectum than colon, more observed cases than expected for both male and female
Levi, 1999 [22]	Vaud Cancer Registry	5261	1974–1994	0.96 (0.4–2.1)	Melanoma SPC more prevalent in colon than rectum
Hemminki, 2001 [15]	Swedish Family-Cancer Database	68,104	1958–1996	1.55 (1.29–1.84)	Included both sporadic and familial CRC, observed cases were greatest at 1–10 years follow-up interval
Dasgupta, 2012 [16]	Queensland Cancer Registry	15,755	1996–2007	1.37 (1.17–1.59)	More observed cases of melanoma than expected cases, melanoma cases more prevalent in males than females, SIR was significant for males but not for females
Lee, 2015 [24]	Taiwan National Health Insurance database	98,876	1996–2011	1.12 (0.93–1.33)	Melanoma SPC more prevalent in colon than rectal cancer, more observed than expected cases in colon but not rectal cancer
He, 2018 [19]	Surveillance, Epidemiology, and End Results database (SEER 8)	44,106	1973–2013	0.89 (0.85–0.95)	Included both young and old patients (< 50, > 50), less observed cases than expected, increasing age led to decreasing risk of SPC development
Ye, 2018 [17]	Tasmanian Cancer Registry	7567	1980–2013	1.36 (1.08–1.70)	Significant SIR for second primary melanoma when first primary CRC was diagnosed between 2000 and 2009
Bright, 2019 [25]	Office for National Statistics, Welsh Cancer Intelligence and Surveillance Unit	5805	1971–2006	1.0 (0.8–1.3)	Cohort is made up of young adult and adolescents (15–39 years old), more observed cases of melanoma than expected cases

### Melanoma and Its Second Primary Cancers

The current literature shows strong evidence for an association between melanoma and the subsequent development of SPCs. A meta-analysis, that analysed over 350,000 melanoma patients from studies worldwide, found an overall increased risk of SPCs following melanoma diagnosis (1.57, 95% CI 1.29–1.90) [11]. In addition, significant risks of colon cancers (SRR 1.12, 95% CI 1.00–1.25), breast cancers (1.14, 95% CI 1.07–1.22), non-melanoma skin cancers (4.01, 95% CI 1.81–8.87), soft tissue tumours (6.80, 95% CI 1.29–35.98), renal cancers (1.34, 95% CI 1.23–1.45), and bone cancers (2.09, 95% CI 1.08–4.05) were also reported [11]. Development of these SPCs could be again due to similar factors—known and possibly unknown risk factors common across different cancers—coinciding in subgroups of patients [11].

#### Colorectal Cancer Following Melanoma

**Fig. 3:**
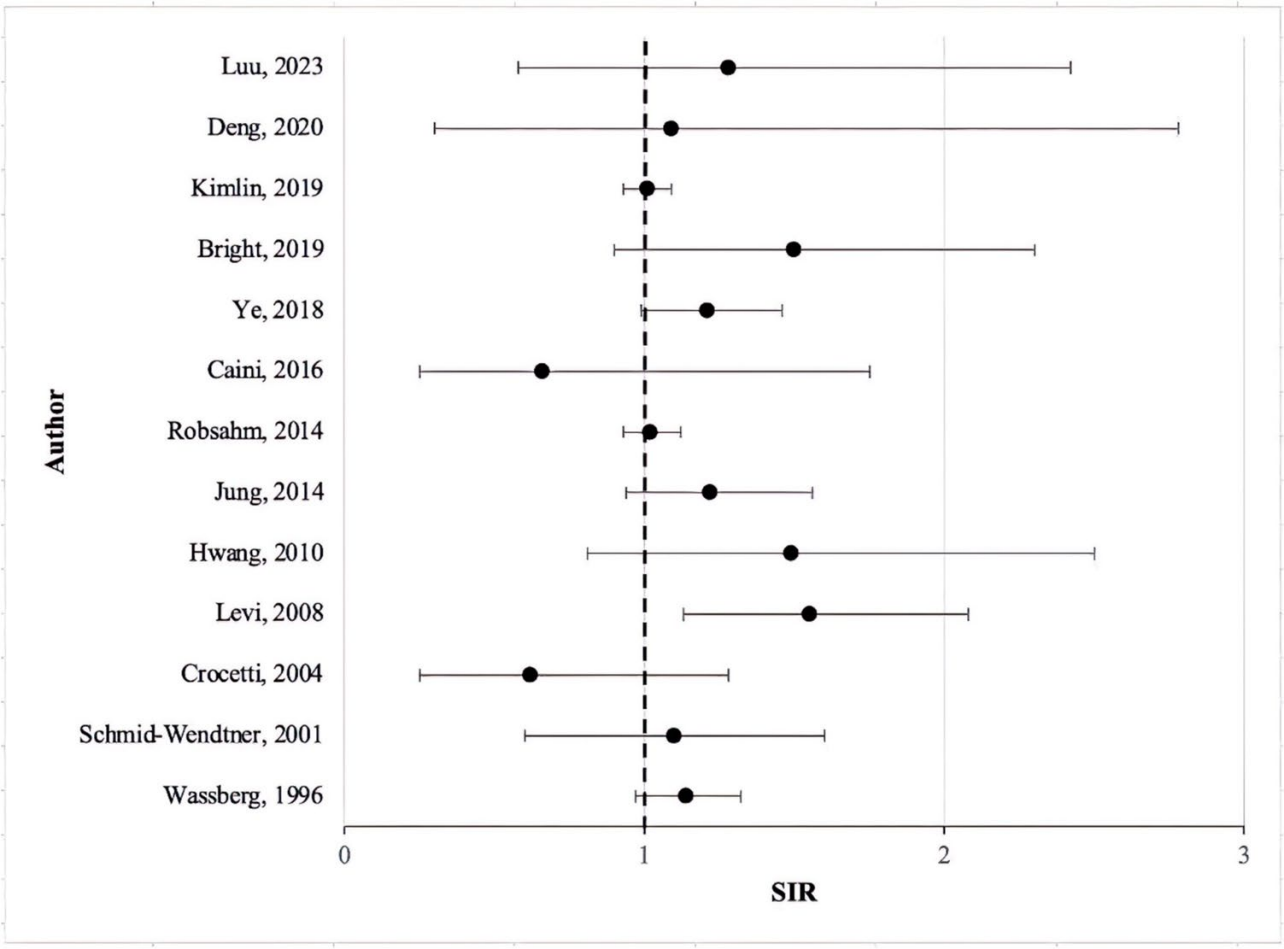
Standardised incidence ratios (SIR) of second primary colorectal cancer following cutaneous melanoma diagnosis. SIR ranged from 0.62 to 1.55 for primary CRC after cutaneous melanoma

Current literature shows strong evidence of CRC in those with a prior diagnosis of melanoma (Table 2). A wide range of SIR reported (0.62–1.55) suggests there was variability across findings, with some papers finding little to no significant increase in risk, while other studies did (Fig. 3). Despite this wider range, most papers reported SIRs above one, indicating an increased risk of second primary CRC in patients with a prior melanoma diagnosis. Only Caini et al. and Crocetti et al. reported a decreased risk of developing CRC, with SIR of 0.66 (0.25–1.75) and 0.62 (0.25–1.28), respectively. However, it should be noted these results were not statistically significant [12, 26].

**Table 2:** Studies investigating the risk of second primary colorectal cancer in patients with a previous melanoma diagnosis

Author, year	Data source	No. of melanoma patients	Span of study	Colorectal cancer SIR (95% CI)	Key findings
Wassberg, 1996 [27]	Swedish Cancer Registry	20 354	1958–1988	1.14 (0.97–1.32)	First primary cancer was cutaneous malignant melanoma (CMM), SPC more prevalent in colon site than rectum, more cases observed for both male and female than expected
Schmid-Wendtner, 2001 [30]	Department of Dermatology and Allergology, Ludwig‐Maximilians‐University, Munich, Germany	4597	1977–1992	1.1 (0.6–1.6)	First primary cancer was CMM, slightly more CRC SPC cases observed for males than expected, 21 patients (0.46%) developed colon cancer as SPC
Crocetti, 2004 [26]	Tuscany Cancer Registry	1835	1985–1999	0.62 (0.25–1.28)	First primary cancer was CMM, colon/rectal SPC risk was increased similarly in both sexes
Levi, 2008 [14]	Vaud and Neuchâtel Cancer Registry	3346	1974–2005	1.55 (1.13–2.08)	Significant SIR in < 60-year-olds, significant SIR for melanomas in sites other than head and neck
Hwang, 2010 [29]	Taiwan’s National Health Insurance Research Database	2665	1997–2008	1.49 (0.81–2.50)	First primary cancer was CMM, increased prevalence of CRC development in males than females
Jung, 2014 [28]	Alberta Cancer Registry	6884	1979–2009	1.22 (0.94–1.56)	First primary cancer was CMM, colon site more prevalent than rectum, SPC less common after CMM than NMSC
Robsahm, 2014 [31]	The Cancer Registry of Norway	28,069	1955–2008	1.02 (0.93–1.12)	First primary cancer was CMM, colon site more prevalent than rectum site, results adjusted for age and calendar period
Caini, 2016 [12]	European Institute of Oncology, Milan Italy	1537	2000–2010	0.66 (0.25–1.75)	First primary cancer was CMM, less colon cases observed than expected, did not find an increased risk of colon cancer SPC development unlike other studies
Ye, 2018 [17]	Tasmanian Cancer Registry	5046	1980–2013	1.21 (0.99–1.46)	More melanoma SPC cases observed than expected, SIR was not significant for any of the calendar periods
Bright, 2019 [25]	Office for National Statistics, Welsh Cancer Intelligence and Surveillance Unit	22,446	1971–2006	1.5 (0.9–2.3)	Cohort is made up of young adult and adolescents (age 15–39 years), more observed cases of CRC than expected
Kimlin, 2019 [32]	Queensland Cancer Registry	39,872	1982–2014	1.01 (0.93–1.09)	First primary cancer was in situ melanoma, increased relative risk of CRC SPC in < 50 years old (at first diagnosis) compared to others, relatively increased risk of CRC SPC development after invasive melanoma than in-situ melanoma
Deng, 2020 [33]	Surveillance, Epidemiology, and End Results database (SEER 18)	5016	2011–2016	1.09 (0.30–2.78)	First primary cancer was metastatic melanoma, smaller number observed than other papers (8 in total over 11 years)
Luu, 2023 [34]	Surveillance, Epidemiology, and End Results database (SEER 18)	7169	2000–2018	1.28 (0.58–2.42)	Study focussed on paediatric and young adult populations (age 0–29 years), similar incidence across sexes

Levi et al. conducted a study with 3346 melanoma patients from Switzerland. A significant SIR of 1.55 (1.13–2.08) was reported, indicating an overall increased risk of developing CRC as an SPC following melanoma. This was statistically significant in those aged less than 60 years old (SIR 1.85, 95% CI 1.08–2.96) but not for those aged more than 60 (SIR 1.42, 95% CI 0.94–2.05), indicating a difference in age at diagnosis [14].

While several studies also reported increased risks, these were not statistically significant [17, 25, 27, 28]. One such study was from Hwang et al., who reported an SIR of 1.49 (95% CI 0.81–2.50). However, a statistically significant increased risk was seen amongst males (SIR 2.05, 95% CI 1.02–3.67) compared to females (SIR 0.76, 95% CI 0.15–2.22) [29].

## Discussion

This review findings suggest that patients diagnosed with melanoma are at a higher risk of developing a second primary CRC. Similarly, CRC survivors have an increased incidence of developing second primary melanoma. This association could be attributed to the shared exposures and risk factors. These risk factors, when present concurrently within the same individual, could elevate the likelihood of developing associated SPCs [35].

### Associated Factors

#### Age

Some studies have suggested that an increasing age may lead to a decreasing risk of SPC development in those with a prior diagnosis of CRC. He et al. reported that overall SIR was inversely associated with the age of onset, and absolute excess risk was increased in the younger populations, particularly those aged 25–45 years old (*p* < 0.05) [19]. Similarly, Levi et al. reported an increased risk of second primary CRC in those who were less than 60 years old [14]. This was corroborated by Kimlin et al., who found that second primary CRC had a significantly greater risk of developing in those less than 50 years old (SIR 1.30, 95% CI 1.05–1.62) compared to those aged 50–69 or more than 70 years old [32]. Therefore, a younger age of melanoma diagnosis may lead to an increased risk of developing CRC as an SPC. Additionally, based on He et al., CRC diagnosis at a younger age may lead to an increased risk of developing subsequent SPCs such as melanoma.

#### Sex

Males are significantly more likely to develop melanoma than females, owing to a lack of preventative behaviours and self-detection of skin lesions. Additionally, research indicates that male skin differs in structure from female skin, with males having thicker skin with less subcutaneous fat, as well as more collagen and elastin. This combination may contribute to an increased likelihood of damage by ultraviolet rays [36]. While the incidence of melanoma is increasing across both sexes, males report a higher rate of 4.4% per year, compared to 3.1% for females [37]. Similarly, an age-adjusted incidence ratio of 1.38 was seen for CRC in males compared to females (*p* < 0.05), indicating a higher CRC incidence among males than females [38]. A study analysing 2665 Taiwanese patients reported an increased risk of CRC development following diagnosis of melanoma. Sex-specific risk increases were noted, with males (SIR 2.05, 95% CI 1.02–3.67) being more than twice as likely to develop CRC compared to females (SIR 0.76, 95% CI 0.15–2.22) [29]. In the development of both melanoma and CRC, male sex appears to be a significant risk factor.

#### Ethnicity

Ethnicity and skin colour have historically been linked to the pathogenesis of certain cancers. A study conducted by Hwang et al. reports that melanoma proneness may be higher in Caucasians than in Asian populations. Therefore, the epigenetic and genetic differences around the pathogenesis of melanoma should also be considered before applying the SIRs [29]. On the other hand, a US-based study reported that non-Hispanic black melanoma patients had a greater SPC risk (SIR 6.65, 95% CI 1.37–19.4) when compared to non-Hispanic white patients (SIR 5.25, 95% CI 4.84–5.69) [34]. Similarly, Chen et al. found that SPC risk was greater in black, male CRC survivors compared to white or other races [39].

Therefore, this suggests ethnicity may be a key risk factor in determining SPC development in primary melanoma or CRC patients.

#### Site of Tumour

A study conducted using 1537 melanoma patients from the European Institute of Oncology in Milan found no increase in developing cancer in the rectum/anus following melanoma incidence, with a SIR of 1.08 (0.27–4.32). Furthermore, a reduced incidence of colon cancer was also noted, with a SIR of 0.47 (0.12–1.89) [12]. However, it should be noted these results were not statistically significant. Additionally, no significant differences were noted by melanoma histology or anatomical sites. The exclusion of in situ melanoma did not affect the SIR for the development of SPCs either [12]. These findings align with Crocetti et al., who conducted a population-based cohort study using cutaneous malignant melanoma patient data from the Tuscany Cancer Registry. A SIR of 0.53 (0.15–1.36) for colon cancer and a SIR of 0.82 (0.17–2.41) for cancer in the rectum were reported, indicating a non-significant reduced risk of second primary CRC development [26]. Taken together, there is no substantial evidence that melanoma may play a role in second primary CRC development in a specific site.

#### Diet

The World Health Organisation (WHO) International Agency for Research on Cancer recently evaluated the carcinogenicity of certain foods. Processed meats—such as those that have been salted, smoked, or cured—were labelled carcinogenic to humans. Red meat was listed as a group 2A substance, indicating its probable carcinogenicity to humans [40].

Red meats contain essential amino acids and other important micronutrients such as iron and B vitamins. However, they also contain saturated fat, which can vary according to the animal and cut of meat. Processing of meat can lead to the formation of carcinogenic chemicals such as polycyclic aromatic hydrocarbons (PAH). The cooking of red meats can also produce PAHs, with frying and grilling resulting in the highest production of these chemicals [41].

Several meta-analyses indicate a weak yet significant association between red/processed meat consumption and CRC incidence, likely due to these chemicals [42–44]. Western diet incorporates the highest proportion of red/processed meats, indicating the greatest increase in risk of CRC development at 31% (RR 1.31, 95% CI 1.15–1.48) [45]. Based on current literature, there is strong evidence for an association between red/processed meat consumption and the development of CRC.

Conversely, Yen et al. reported an inverse association between melanoma risk and red/processed meat consumption. A total of 1318 melanoma patients were studied during follow-up, resulting in pooled hazard ratios of 1.00 (0.87–1.14), 0.98 (0.86–1.13), 0.89 (0.77–1.02), and 0.81 (0.70–0.95) for increasing quintiles of intake [46]. However, the link between diet and melanoma development is inconsistent in recent literature. Rothberg et al. noted that the consumption of red meat at least once a week was associated with lower survival in melanoma patients (HR 1.84, 95% CI: 1.02–3.30) [47]. Whilst the evidence for the role of diet in CRC is well established, whether certain dietary molecules contribute to cutaneous melanoma pathogenesis needs further investigation.

#### Alcohol

Alcohol is reported as carcinogenic due to its ethanol content. This can increase levels of acetaldehyde in the body, resulting in DNA damage [48]. While the type of alcohol is inconsequential, moderate consumption (defined as 1–2 drinks/day) may contribute to a higher risk of some cancers, including CRC [49]. One meta-analysis found an increased association between moderate alcohol consumption and development of CRC, significantly greater in males (RR 1.24, 95% CI 1.13–1.37) than in females (RR 1.08, 95% CI 1.03–1.13) [50]. However, Kim et al. found that light-moderate pre-diagnostic alcohol consumption was associated with better CRC survival [51]. Nevertheless, recent literature shows greater support for a dose–response association between alcohol intake and increased risk of CRC [52–56].

While alcohol intake is also associated with cutaneous melanoma, it is credited to an increase in sunburn severity. This is because alcohol causes a reduction in the protective efficacy of antioxidants [57]. Additionally, baseline alcohol intake led to a non-significant increase in melanoma development in males (HR 1.17, 95% CI = 0.95–1.44), to a greater degree than in females (HR 0.93, 95% CI = 0.80–1.08) [58]. While several meta-analyses also supported the association between cutaneous melanoma and alcohol intake, each reported that residual confounding and bias could not be ruled out [59–63]. While there is strong evidence for alcohol and the risk of CRC, further research may be needed to confirm links with melanoma.

#### Environmental Factors

Increased sun exposure has repeatedly been linked with a higher incidence of cutaneous melanoma [64–67]. Several physical characteristics—such as freckles, fairer skin tone, and hair colour—may also contribute to this association. Due to variations in patterns of sun exposure, factors such as lower latitude and anatomical tumour site are also seen to play a role in determining melanoma risk [68, 69]. Physiological vitamin D derivatives, activated through sun exposure, are seen to have antiproliferative, anti-inflammatory, and anticancer properties. Therefore, defects in vitamin D signalling pathways may have a role in the propagation of melanoma. Defective activation/inactivation of vitamin D and corresponding receptors has been seen to affect the progression and outcome of cutaneous melanoma development [70].

Alternatively, sun exposure, and in turn, vitamin D, has been linked with a protective effect in CRC [14, 71–73]. In 1980, the Garland brothers proposed a hypothesis for this. Vitamin D, produced from cholesterol after sunlight exposure, promotes intestinal absorption of calcium. Once entering the circulation, calcium reduces tissue reactivity to inflammatory stimuli in the gut, exhibiting a protective effect against CRC [74]. Therefore, sun exposure and vitamin D may have opposing roles in the development of melanoma or CRC.

#### Genetics

While unshared environmental factors account for 67–68% of sporadic cases of melanoma and colorectal cancer in the general population, making them the most significant contributors, 10–18% can still be attributed to genetic factors [75]. Specific gene mutations, such as KRAS, NRAS, and BRAF, are known to play a crucial role in the propagation of both cancers [76]. Additionally, mutations in BRCA2 and CDKN2A have been associated with the pathogenesis of colorectal cancer. These key genetic factors and their functional significance in SPC development are detailed below.

##### *KRAS*

KRAS is part of the RAS superfamily, which consists of small GTP-binding proteins. It relays external signals to the cell’s nucleus, enabling its role as a regulatory protein in cell division [77]. If mutations arise in the *KRAS* gene, this may lead to problems switching between active and inactive states for the KRAS protein, leading to cell transformation and carcinogenesis. Therefore, these mutations can act as diagnostic biomarkers [78].

As the most common, 40% of CRCs are estimated to exhibit missense mutations in the *KRAS* gene [79]. The frequency of these mutations is said to be dependent on the grade of the tumour, with grade 1–2 displaying a higher frequency of mutations than grade 3–4 tumours. Additionally, microsatellite instability also depends on the grade, with frequency increasing in lower and more stable tumours [80]. Compared to CRC, *KRAS* mutations account for only 2% of melanoma cases, and the reason for this remains unknown [81]. Therefore, while *KRAS* mutations can be present in both types of cancers, they may be more prevalent in CRC development.

##### *BRAF*

The BRAF protein is involved in the MAPK signalling pathway, responsible for cell proliferation and differentiation. Therefore, a mutation in this gene can lead to overstimulation of the protein and inappropriate growth of cells [82]. Compared to *KRAS* mutations, *BRAF* is less frequent in CRCs, at 11% [83]. However, right-sided and high-grade tumours are more frequently associated with *BRAF* mutations [80]. In contrast, *BRAF* mutations are the most common for cutaneous melanomas, making up approximately 50–60%. These mutations occur in 80% of melanocytic nevi, indicating changes early in melanoma pathogenesis [84]. Within both melanoma and CRCs, the most common form of mutation is *BRAF V600E* [85]. Like *KRAS* mutations, *BRAF* can be present in both cancers but appears to be more prevalent in melanoma development than in CRC development.

##### *NRAS*

*NRAS*, neuroblastoma-RAS, is one of the three RAS genes involved in the crossroads between cell surface receptors and internal cellular processes, leading to cell proliferation, differentiation, and apoptosis [86]. *NRAS* mutations are seen only in 2–4% of CRCs, unlike *KRAS* or *BRAF* mutations [87]. On the other hand, *NRAS* mutations are more common for cutaneous melanomas, occurring in 10–15% of melanomas [88]. *NRAS* mutations are typically associated with higher rates of mitosis and thicker tumours compared to *BRAF V600E* [89]. Unlike *KRAS* and *BRAF* mutations, *NRAS* mutations may be more significant in melanoma than CRC, but can still be seen in both cancers.

##### *BRCA*

*BRCA1* and *BRCA2* are tumour suppressor genes responsible for homologous recombination repair of DNA lesions, particularly double-stranded DNA breaks. If these genes were to be mutated, chromosomal stability would be compromised, increasing the risk of carcinogenesis [90]. *BRCA1* is not significantly associated with cutaneous melanoma, whereas *BRCA2* is (RR 2.58, 95% CI 1.28–5.17) [91]. However, a systematic review conducted by Cullinane et al. concluded that *BRCA2* carriers are not at a higher risk for CRC development despite a positive family history being one of the most critical CRC risk factors [92]. Interestingly, both Oh et al. and Phelan et al. reported an increased risk of CRC in *BRCA1* mutation rather than *BRCA2* [93, 94]. However, Gay-Bellile et al. found that biallelic *BRCA2* variants may be implicated in familial CRC inheritance, though further studies would be needed to confirm this association [95]. Similarly, Vikstrom et al. reported insufficient evidence to conclusively link between *BRCA2* and CRC pathogenesis [96]. While there is evidence for melanoma development, there appear to be mixed findings for *BRCA2* involvement in CRC, with further research being needed to confirm either stance.

##### *CDK2NA*

Cyclin-dependent kinase inhibitor 2A (*CDK2NA*) produces the tumour suppressor protein, p16, which is responsible for inhibiting the kinase activity of CDK4 and CDK6. Under physiological circumstances, this slows the progression of the cell cycle to prevent excessive replication. However, homozygous *CDK2NA* mutations or deletions may be responsible for increased melanoma susceptibility [97]. In contrast, neither *CDK2NA* (p16) nor *CDK2NA* promotor methylation was independently associated with CRC patient prognosis [98]. On the other hand, a meta-analysis conducted by Xing et al. reported that *CDK2NA* hypermethylation may be a predictor factor for poor prognosis in CRC patients [99]. Similarly, a cohort study suggested that high to moderate levels of p16 expression in CRC sections may indicate that p16 plays a role in CRC carcinogenesis—though further research, with larger sample sizes, will be needed to confirm these findings [100]. Like *BRCA2*, evidence in the current literature is inadequate to confirm the role of *CDK2NA* mutations in CRC development.

### Limitations

The primary limitation of this study is the possibility of missing relevant papers in the initial search. However, given the diversity of included studies, conducted over a broad range of years, we believe that any missed papers are likely few and would not significantly impact our results. Another limitation is the inclusion of studies spanning a wide time range and originating from various countries. This introduces potential confounders, such as differences in cancer screening protocols across countries and how they have evolved over time, which are not accounted for in this review. Nonetheless, we deemed it important to offer a global perspective, tracing back to the earliest published links between melanoma and CRC.

#### Surveillance Bias

Improvements in cancer screening and surveillance may have contributed to increased incidence of SPCs, along with the associations seen between melanoma and CRC, in recent years. The incidence of these malignancies is typically higher in populations of a higher socioeconomic status, which may be due to excellent screening participation and education surrounding risk factors of cancer [101]. Additionally, a patient’s initial cancer diagnosis leads to numerous hospital visits, tests, and long-term follow-ups during which subsequent cancers can be detected [102]. This may be particularly relevant in earlier detection of slow-growing cancers, such as specific subtypes of CRC, contributing to length time bias [103]. Patients with prior diagnoses of cancer are also provided with more education, leading to proactivity in reporting worrisome symptoms. As these patients have more frequent access to the healthcare system, this can lead to potential confounding by surveillance bias. This has been highlighted in a recent study that found the incidence of SPCs is common among patients within 2 years of their initial melanoma diagnosis, likely due to follow-up being most intense during this time [12]. Similarly, a meta-analysis conducted by Caini et al. reported that the overall risk of SPC development decreased as the timeframe from the initial melanoma diagnosis increased [11].

## Conclusion

As survival rates for both melanoma and CRC improve, the risk of SPCs is also rising. This review demonstrated that melanoma survivors have an increased risk of second primary CRC, with SIRs ranging from 0.62 to 1.55. Similarly, CRC survivors showed an increased incidence of developing a second primary melanoma, with SIRs ranging from 0.89 to 1.55. The association between melanoma and CRC is influenced by various biological, lifestyle, and genetic factors. The review identified several key biological risk factors associated with increased incidences of melanoma or CRC. These include male sex, younger age, and African-American populations, all of which reported higher rates of these cancers. Significant lifestyle factors common to both cancers were consumption of red/processed meats, alcohol intake, and sun exposure. Genetic mutations in *KRAS*, *NRAS*, and *BRAF* were frequently observed in both melanoma and CRC, whereas BRCA2 and CDKN2A mutations were specific to melanoma. However, some studies reported inconsistent findings regarding these factors, which may be attributed to differences in data collection methods or variations in the baseline characteristics of the study populations. Identifying these common risk factors could provide a basis for developing future surveillance guidelines for melanoma or CRC survivors. Further research into clinical correlations and shared risk factors between these cancers could enhance screening strategies for SPCs, potentially allowing health practitioners to better predict SPC risk and improve survivability.

## Data Availability

No datasets were generated or analysed during the current study.
